# Supplementary material for: 3D markerless tracking of speech movements with submillimeter accuracy
Source: bioRxiv. 2025 Feb 16:2025.02.13.638009. Preprint. [Version 1] doi: 10.1101/2025.02.13.638009 (PMC11844461; doi:10.1101/2025.02.13.638009)
Supplement: 1 [file NIHPP2025.02.13.638009V1-supplement-1.pdf]

bioRxiv preprint doi: https://doi.org/10.1101/2025.02.13.638009; this version posted February 16, 2025. The copyright holder for this preprint (which was not certified by peer review) is the author/funder, who has granted bioRxiv a license to display the preprint in perpetuity. It is made available under aCC-BY 4.0 International license.

Supporting information

S1 Fig.

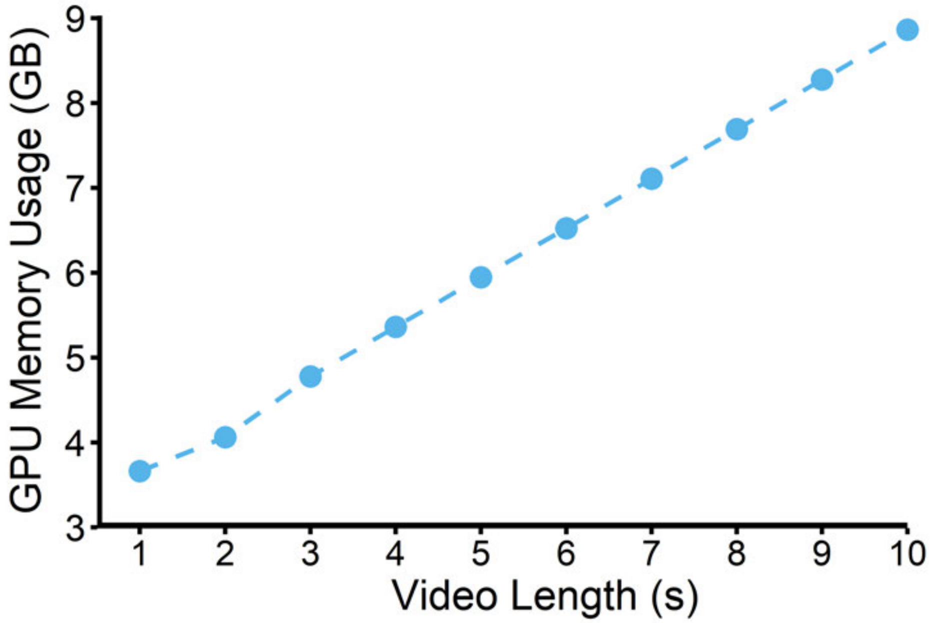

GPU memory usage. CoTracker was used to extract lip landmarks from different time lengths of the same video (*i.e.*, the first second, the first two seconds, and so on,) and the total amount of GPU memory usage on the computer (including the operation system processes) was recorded. The relationship between GPU memory and the video length was highly linear. Even for a 10 second-long video, the required GPU memory is around 10 GB.
